# Delimiting cryptic morphological variation among human malaria vector species using convolutional neural networks

**DOI:** 10.1371/journal.pntd.0008904

**Published:** 2020-12-17

**Authors:** Jannelle Couret, Danilo C. Moreira, Davin Bernier, Aria Mia Loberti, Ellen M. Dotson, Marco Alvarez

**Affiliations:** 1 Department of Biological Sciences, University of Rhode Island, Kingston, Rhode Island, US; 2 Department of Computer Science and Statistics, University of Rhode Island, Kingston, Rhode Island, US; 3 Department of Computer Science, Federal University of Campina Grande, Campina Grande, Brazil; 4 Centers for Disease Control and Prevention, Center for Global Health, Division of Parasitic Diseases and Malaria, Atlanta, Georgia, US; Vienna, AUSTRIA

## Abstract

Deep learning is a powerful approach for distinguishing classes of images, and there is a growing interest in applying these methods to delimit species, particularly in the identification of mosquito vectors. Visual identification of mosquito species is the foundation of mosquito-borne disease surveillance and management, but can be hindered by cryptic morphological variation in mosquito vector species complexes such as the malaria-transmitting *Anopheles gambiae* complex. We sought to apply Convolutional Neural Networks (CNNs) to images of mosquitoes as a proof-of-concept to determine the feasibility of automatic classification of mosquito sex, genus, species, and strains using whole-body, 2D images of mosquitoes. We introduce a library of 1, 709 images of adult mosquitoes collected from 16 colonies of mosquito vector species and strains originating from five geographic regions, with 4 cryptic species not readily distinguishable morphologically even by trained medical entomologists. We present a methodology for image processing, data augmentation, and training and validation of a CNN. Our best CNN configuration achieved high prediction accuracies of 96.96% for species identification and 98.48% for sex. Our results demonstrate that CNNs can delimit species with cryptic morphological variation, 2 strains of a single species, and specimens from a single colony stored using two different methods. We present visualizations of the CNN feature space and predictions for interpretation of our results, and we further discuss applications of our findings for future applications in malaria mosquito surveillance.

## Author summary

Rapid and accurate identification of mosquitoes that transmit human pathogens is an essential part of mosquito-borne disease surveillance. Such identification can be difficult for mosquitoes that transmit malaria, as many are morphologically indistinguishable, including those in the *Anopheles gambiae* species complex. We photographed 1, 709 individual mosquitoes from 16 laboratory colonies housed at the Centers for Disease Control and Prevention to create a database of whole-body mosquito images. We present a methodology for image processing, data augmentation, and training and validation of a convolutional neural network (CNN). We applied this method to our mosquito image database, finding a 96.96% prediction accuracy for class identification and 98.48% for sex. Further, our best model accurately predicted images between 2 strains of a single species and between 2 storage methods of mosquitoes from the same colony. These results demonstrate that image classification with deep learning can be a useful method for malaria mosquito identification, even among species with cryptic morphological variation. We discuss the application of deep learning to mosquito identification in malaria mosquito surveillance.

## Introduction

Human malaria is an ongoing public health crisis affecting multiple continents, with the highest numbers of cases and people at risk occurring in sub-Saharan Africa [1, 2]. There, the principal vectors of *Plasmodium falciparum* malaria are members of a species complex called *An*. *gambiae* sensu lato [3, 4]. These sibling species are nearly indistinguishable morphologically, though genetically divergent [5, 6]. Even among highly trained taxonomists specializing in the species complex, member species cannot be reliably distinguished on the basis of morphology alone. Yet the identification and delineation of the *Anopheles* genus is recognized as a critical step in the surveillance and control of malaria [7, 8].

Taxonomic keys have been developed and are widely used for species with conspicuous morphology [9], but cannot be used to distinguish among *An*. *gambiae* sensu lato. Member species in the *An*. *gambiae* complex commonly occur in sympatry across several African countries with evidence of temporal overlap in peak abundance [10]. Although *An*. *gambiae* complex members largely retain their species identity through hybrid male sterility [11], there is evidence of some gene flow between them [12]. Complex members also vary in several traits expected to influence vectorial capacity including larval ecology, adult habitat, behaviors that impact bloodfeeding such as resting location, and human biting [13–15]. The barrier of morphological identification of species within the *An*. *gambiae* complex remains problematic for effective mosquito vector surveillance, management, and control in some areas. Identification by molecular markers using polymerase chain reaction (PCR) is therefore currently the standard protocol in the surveillance and control of human malaria in Africa, and field collections are, in practice, only identified using laboratory testing of molecular markers.

### Machine learning applications to mosquito-borne disease

There is a burgeoning interest in the application of machine learning techniques to address the emerging and reemerging public health threats of mosquito-borne diseases. Machine learning has been applied to the forecasting of mosquito-borne disease incidence [16–18], to model mosquito habitat quality [19], to identify and quantify breeding sites [20], to find patterns in mosquito surveillance data including invasive species [21, 22], and, most recently, in the automatic identification of mosquito species. Machine learning with acoustic classification of wingbeat frequency has been explored to determine mosquito genus [23–26]. Visual methods have also been explored for mosquito genus and species identification by extracting features from images before applying supervised machine learning techniques such as Support Vector Machine (SVM) learning. Extracted features vary between studies but have included color histograms of images, length of body and legs, and wing length and shape [27]. SVM learning of images of specific body parts such as the wings, proboscis, palps, or scuta has been applied by others to targeted identification of medically important species such as *Aedes aegypti* and *Aedes albopictus* [28–30]. The methods of applying machine learning to mosquito identification are now progressing toward deep learning techniques, and there is a growing interest in applying these methods to delimit species [31, 32], particularly in the identification of mosquito vectors. This change in methodology corresponds to a shift in the biological questions that can be considered as well. Whereas SVM can determine whether an insect is or is not a mosquito [33], and whether a mosquito is or is not *Ae*. *aegypti* [28, 29], deep learning can be applied to distinguish between many mosquito species. The applications of these methods are well suited for the study of morphological variation between and within species.

Convolutional Neural Networks (CNNs) have been successfully applied to a broad range of image datasets to separate images into classes and accurately predict class when presented with novel images, in some cases even surpassing human performance in specific tasks [34]. While other methods applying machine learning to mosquito identification have required manually extracted features as inputs, CNNs can learn from images by directly analyzing pixels. It is important to note that in the discipline of machine learning ‘class’ refers to the label category and does not directly relate to the biological taxonomic definition. CNNs are of growing interest to distinguish mosquito genera and species relating to other mosquito-borne pathogens. Ortiz *et al*. applied a CNN to vision-based identification of larval *Aedes* mosquitoes, with images of the 8th segment comb scales on fourth instar larvae [35]. CNNs have been applied to distinguish fourth instar from pupa in *Ae*. *aegypti* [36], though the need of such a model from a biological perspective is questionable as these life stages are readily distinguishable. Most recently, Park et al. [37] trained a CNN with whole body mosquito images collected from field sites to distinguish between 8 local species in Japan, of which some are vectors of human pathogens, across three genera. A commonality among these studies is that these methods were applied to distinguish species with known and conspicuous morphological differences evident to the human eye. Indeed, CNNs have been previously applied to classify species with known morphological differences across diverse taxa [31, 32, 38, 39].

### Delimitation of cryptic morphological variation with machine learning

The application of machine learning to distinguish between species with cryptic morphological variation has been controversial. Indeed, recent reviews on the topic of delimiting cryptic species [40] and cryptic malaria vectors in Africa [3] have largely dismissed machine learning for not having addressed important biological questions in taxonomy. In addition, few studies have applied machine learning to *Anopheles* mosquitoes relative to other genera of mosquito vectors. One notable exception is work by González Jiménez *et al* which compared several machine learning approaches to distinguish two Anopheles mosquitoes, *Anopheles gambiae* and *Anopheles arabiensis* known to have cryptic variation [41]. The highest prediction accuracy to distinguish the two species was 82.6%, the result of bagging the best logistic regression models. An unanswered question is whether automatic image classification with deep learning can be applied to distinguish among many species known to have cryptic variation in morphology.

We present the results of proof-of-concept experiments in training CNNs on a dataset of whole-body, 2D images to distinguish: *Anopheles* from other mosquito genera, species within *Anopheles*, cryptic species within the *An*. *gambiae* complex, two strains of single species, and two storage methods of mosquitoes from the same colony. We sought to demonstrate that CNNs can distinguish malaria vector species, including those with cryptic morphological variation, using image data with minimal image processing prior to model training. The image dataset of human malaria vectors created in this work can assist in the future training of region-specific models to identify malaria vectors in applied settings.

## Methods

### Malaria mosquito image dataset

As publicly available mosquito images are generally limited to a few images per species, we created a novel image dataset of mosquitoes sampled live from established colonies maintained. Mosquito colonies are maintained by the Malaria Research and Reference Reagent Resource (MR4) / Biodefense and Emerging Infections (BEI) Resources at the Centers for Disease Control and Prevention (CDC) in Atlanta, GA. The MR4/BEI repository of mosquito colonies has the advantage of being one of the few which authenticates species and strain every five generations. We explore the application of different CNN architectures to whole-body images of mosquito vectors of human malaria across sourced from populations in Africa, the Asia-Pacific region, Australia, South America, Caribbean, Europe, and North America (Table 1).

**Table 1:** Mosquito species and number of male and female imaged.

	Female	Male	Origin	Continental Range
*Anopheles arabiensis* *	126	67	Sudan	Africa
*Anopheles coluzzii* *	52	22	Mali	Africa
*Anopheles gambiae s.s*. *KISUMU1* *	76	41	Kenya	Africa
*Anopheles gambiae s.s*. *G3* *	32	21	Gambia	Africa
*Anopheles merus* *	24	26	South Africa	Africa
*Anopheles funestus*	75	62	Mozambique	Africa
*Anopheles faurati*	11	46	Papua New Guinea	Asia-Pacific, Australia
*Anopheles minimus*	54	64	Thailand	Asia-Pacific region
*Anopheles stephensi* (dried)	102	62	India	Asia-Pacific region
*Anopheles stephensi*	26	27	India	Asia-Pacific region
*Anopheles albimanus*	40	24	El Salvador	South America, Caribbean
*Anopheles atroparvus*	36	0	Spain	Europe
*Anopheles freeborni*	45	52	USA	North America
*Anopheles quadrimaculatus*	42	5	Unknown	North America
*Culex quinquefasciatus*	48	5	South Africa	All, sub-tropical
*Culex tarsalis*	130	47	USA	North America
*Aedes aegypti*	151	68	Costa Rica	All, tropical, sub-tropical

(*) indicates that the species is a member of the *Anopheles gambiae* species complex.

By sampling laboratory colony mosquitoes, we were able to access more malaria mosquito vector species over a wider geographic range than would be feasible within one study with field trapping of mosquitoes. This was preferred in order to include substantial morphological variation within the genus to test deep learning techniques. However, mosquitoes in colony have the potential to diverge to varying extents in morphology from field populations because of the restricted gene flow in colony relative to wild populations. Morphometric analysis of multiple characters has been successful at distinguishing field and laboratory strains of *An*. *arabiensis* and *An*. *gambiae* s.s. [42], albeit with error rates nearing 10%. We therefore present this as a proof-of-concept that deep learning can be applied to *Anopheles* mosquitoes, particularly including those with cryptic variation, rather than field-ready tool for identification of images of wild mosquito populations.

Adults, both males and females, were imaged from 15 species of mosquitoes, 13 from the genus *Anopheles*, 2 from *Culex* and 1 from *Aedes*. We included 2 strains of *An*. *gambiae s.s*., G3 and KISUMU1, and 2 methods of storing *An*. *stephensi* resulting a total of 17 classes. The ages of individual mosquitoes varied by several weeks, but all females had blood-fed and produced eggs in at least one gonotrophic cycle. All mosquitoes had constant access to a 10% sugar solution while in colonies prior to freezing and imaging. The total number of images was 1, 709 and the number of images per category is summarized in Table 1. At least one strain from each species of malaria vectors housed in colony at MR4 is represented in the dataset, resulting in a representation of malaria vector species across several global regions as shown in Table 1. Mosquito images were labeled by genus, species, strain, and sex. In addition, we captured images of *An*. *stephensi* that had been stored in two different ways, flash freezing versus dried. We sought to test whether mosquitoes from the same colony could be distinguished solely on the basis of storage method.

**Fig 1:**
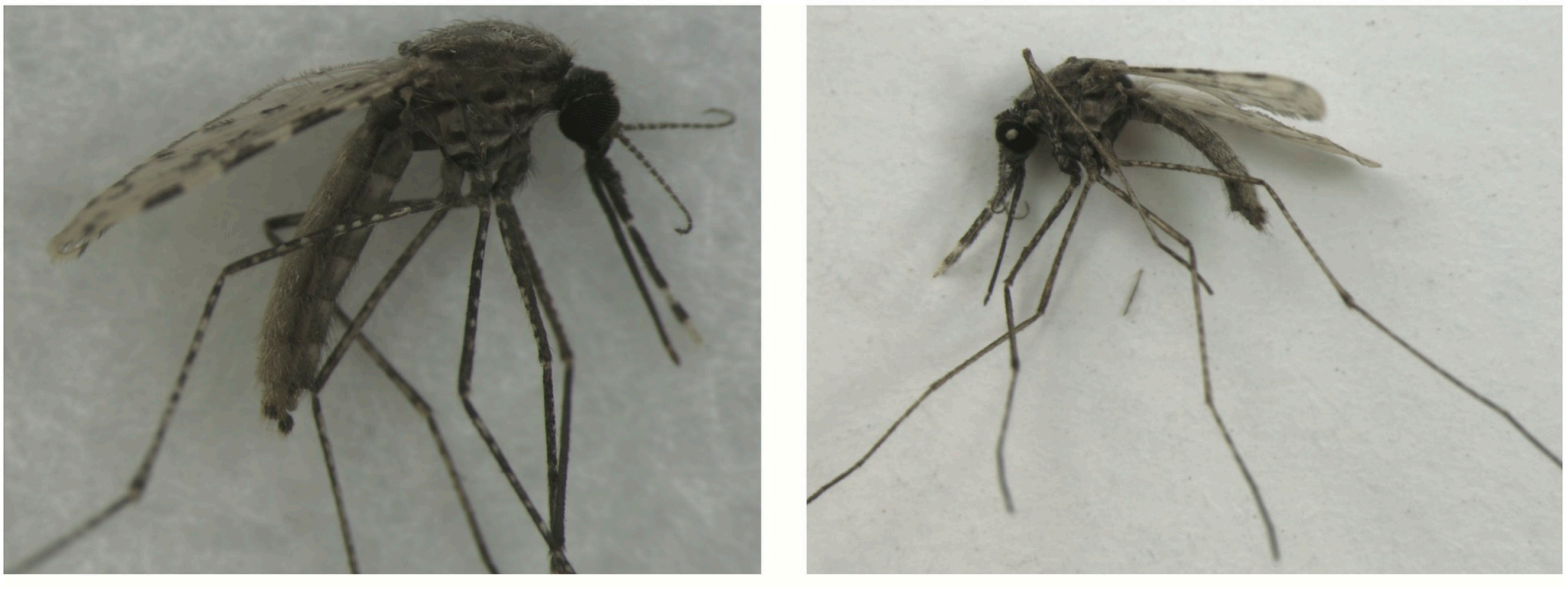
Representative specimen images of *Anopheles stephensi*. Picture on the left shows a mosquito stored by freezing at -80C. Picture on the right shows a mosquito desiccated at room temperature.

For the 16 classes of mosquitoes that were not dried, they were collected from colony cages and placed in a −80°C celsius freezer for a minimum of 20 minutes prior to use. Dried mosquitoes were allowed to desiccate for at least 48 hours prior to use. Fig 1 shows a visual comparison of specimens stored by flash freezing versus those desiccated.

**Fig 2:**
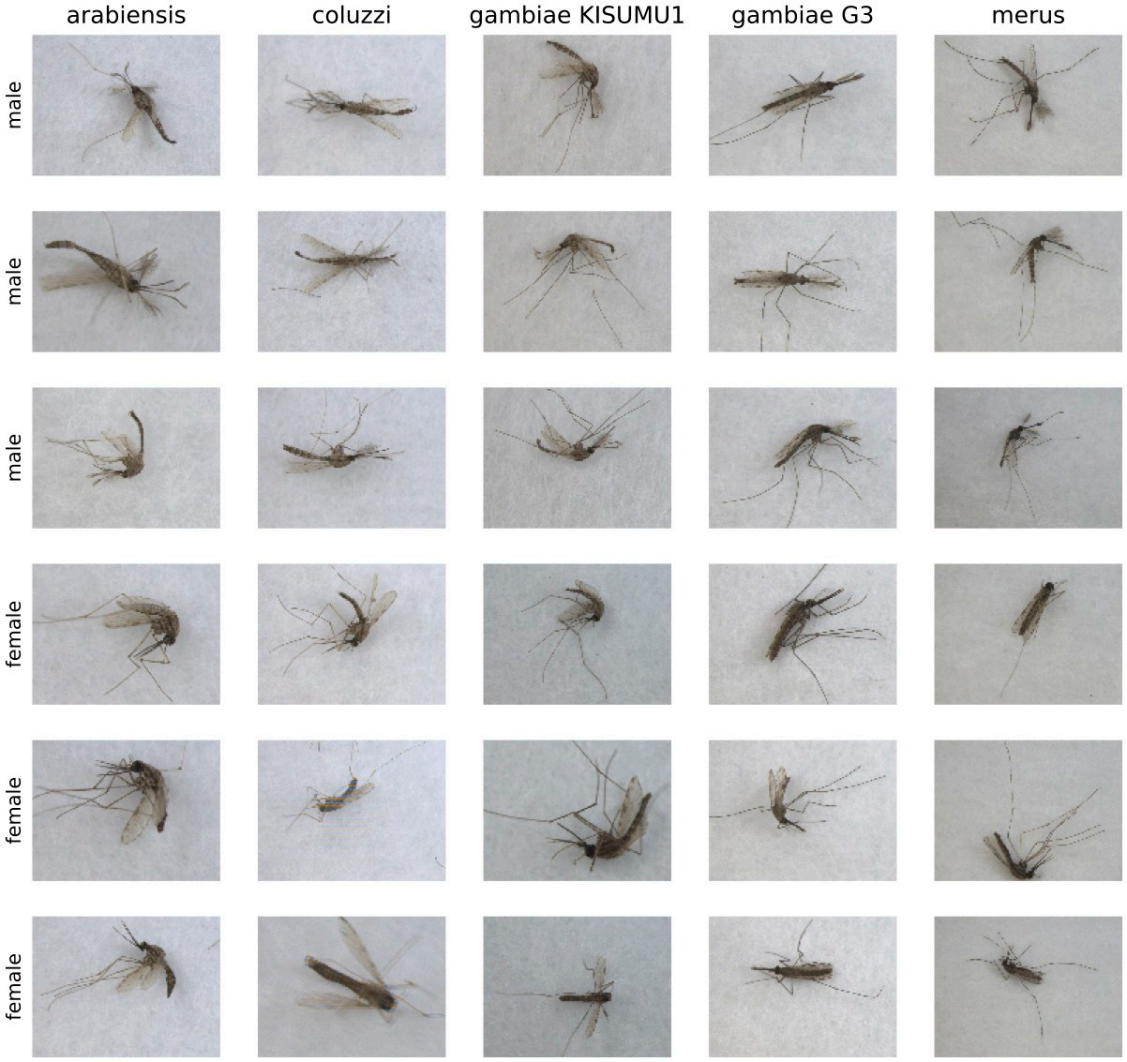
Sample images from our image dataset. For each species member of the *An*. *gambiae* complex, 3 random females and 3 random males are displayed.

Images were captured using a stereomicroscope (Leica M205) with an attached 10-megapixel digital camera. A sheet of plain white paper was placed between the light source and the field in order to diffuse the light and reduce shadows in the images. Photos captured were of individual mosquitoes such that the total number of images matches the total number of specimens photographed per category and sex. In order to increase the variability of the photos collected and allow for a more robust computational analysis, each mosquito during sampling was oriented randomly to capture the ventral, dorsal, or lateral view. This was easy to accomplish with fresh specimens and only slightly more difficult to accomplish with the dried *An*. *stephensi* specimens. The latter tended to fall to the lateral or ventral view, requiring additional time to position them dorsally. Both magnification (10x, 20x, 30x, 40x) and depth of focus were deliberately varied between images to increase image diversity. Some images capture the entirety of the specimen including all leg segments, and others cropping out portions of the legs. Because the depth of focus varied, some images appear to be in less focus than others, though at least one body part within the image is in focus. Fig 2 shows a sample of male and female mosquitos from the *An*. *gambiae* complex.

### Dividing data into training, validation, and test sets

**Fig 3:**
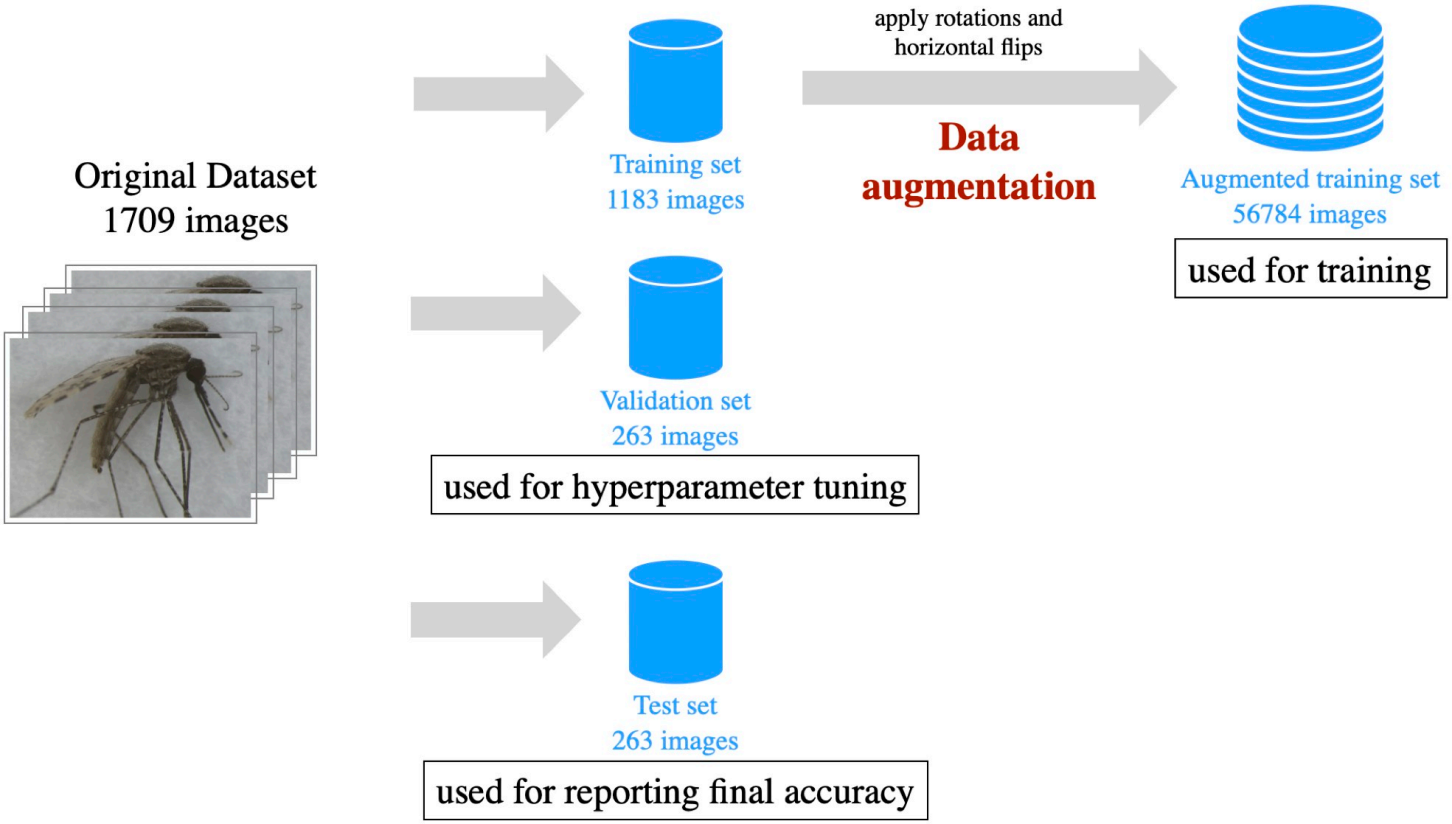
Dataset preparation for applying machine learning. The original dataset of images was split into three partitions, training, validation and test.

All images from the original dataset were annotated with one of the 17 categories show in Table 1. To make sure all categories were well represented, we used a stratified approach. The data were partitioned randomly into the training, validation, and test sets, comprised of 70%, 15%, and 15% of the dataset respectively as shown in Fig 3. The training set is used to learn relevant feature representations from the images by adjusting the internal weights of the classes in the CNN. The validation set is used to evaluate the performance of the CNN during the training process. In addition, the validation set determines the best configuration of hyperparameters that control the learning process. The sole purpose of the test set is to provide a fair reporting of final performance, therefore the images from the test set are not used at any stage of the training process. Fig 3 shows further details on the partition of the original dataset.

### Pre-processing and data augmentation

**Fig 4:**
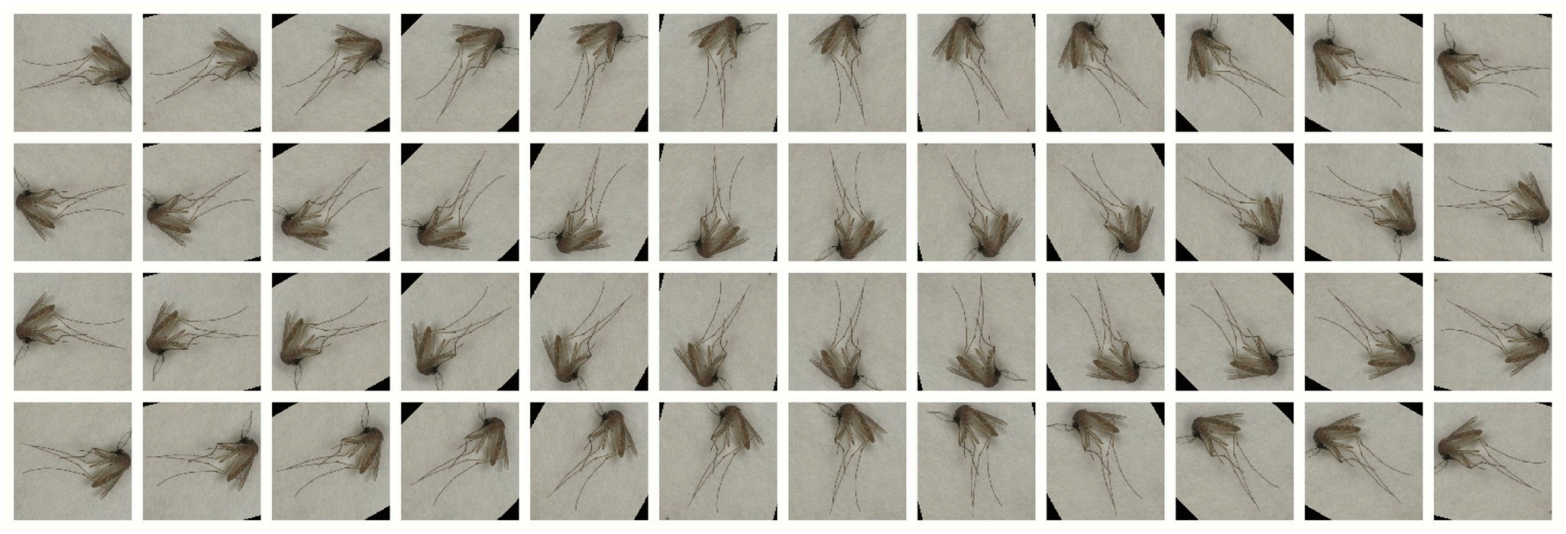
Data augmentation applied to a single image. 48 images synthetically generated (augmented) from an input image. The first 2 rows show 24 rotations applied to the original image and the last two rows show the 24 rotations applied to a horizontal flip of the original image.

Achieving satisfactory results of high prediction accuracy with CNNs relies on the availability of sufficiently large datasets for the training process. We sought to increase the overall size of the training set as well as the diversity of images within it, applying a technique called *data augmentation*. We were able to substantially increase the size and diversity of the training set of images by using basic transformations such as rotations, flips, resizing, and cropping. We applied 24 rotations with increments of 15 degrees, to both the original image and its horizontal flip. Images were further resized to a length of 256 pixels on its shortest axis, while preserving the aspect ratio. Finally, images were center-cropped to a size of 224 by 224 pixels. With rotations, small triangular regions were introduced in the background of some images. However, these black regions do not play a role in the quality of the trained CNN model. Fig 4 shows examples of the images synthetically generated from a single mosquito image.

Data augmentation strategies are widely accepted and used by deep learning practitioners [43]. Used properly, data augmentation can improve the generalization capabilities of the trained model, reducing overfitting (i.e. memorizing) of the training images. The problem of pseudoreplication was avoided by applying data augmentation only to the images in the training set. This prevents contamination of the test set which is used for assessing the quality of the predictions. Table 2 shows the final number of images on each of the partitions, after data augmentation.

**Table 2:** Split of the original data into three different partitions.

Partition	Number of Images	Percentage
Training Set	1, 183	≈ 70% of original data
Validation Set	263	≈ 15% of original data
Test Set	263	≈ 15% of original data
Augmented Training Set	56, 784	4800% of the training set

### CNN model configuration and training

CNNs consist of several ‘layers’ chained together sequentially with the goal of automatically extracting features. Each layer can perform multiple operations, including applying convolutions, downsampling, or applying linear transformations to its inputs. These operations can be coupled with activation functions to introduce nonlinearities, resulting in neural networks that have dramatically improved the state-of-the-art in speech recognition, computer vision, and many other domains [44].

**Fig 5:**
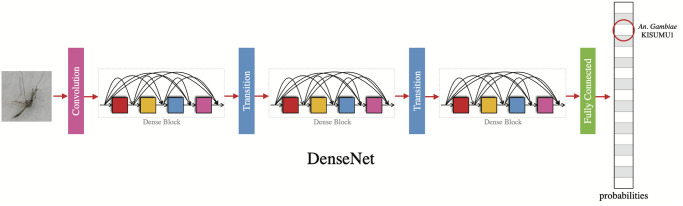
Dense convolutional network (DenseNet). In this architecture each layer is connected to every other layer within the dense blocks in a feed-forward fashion. DenseNets improve gradient-flow during training, strengthen feature propagation, and substantially reduce the number of weights. The input to the network is an image from the dataset and the output is a vector of probabilities for each of the 17 classes.

We evaluated four different CNN architectures for image classification, all variants of the ResNet (residual network) [45], including ResNet [45], Wide ResNet [46], ResNext [47], and DenseNet [48]. These architectures are widely-known due to their successful performance in competitions in the field of computer vision such as the Large Scale Visual Recognition Challenges (ILSVRC) [34]. Fig 5 shows an illustration of the DenseNet, the architecture with the highest performance in our experiments.

The fine tuning of different configurations of hyperparameters is one of the most daunting tasks for machine learning practitioners, as it is time consuming and has a direct influence on the quality of the trained models. We employed a straightforward experimental approach of searching the space of different network architectures, batch sizes, optimizers, and learning rates, in that order. Although this strategy does not guarantee optimality, it is a simple and efficient search with superlative results. The configuration that achieved the highest accuracy was comprised of a DenseNet with 201 layers, batch size of 128, ASGD optimizer, and a learning rate of 2^−2^. These details of hyperparameter selection are necessary to reproduce our results, and further details of hyperparameter selection are presented alongside results.

The CNN weights and biases (i.e. the ‘learnable’ parameters) were initialized from a pre-trained model, a standard practice called *transfer learning* that begins the learning and training process from the weights of a much larger image dataset with over one million images from 1000 classes, called ImageNet [49]. ImageNet does not include mosquitoes, but it is widely used in CNN applications to capture low-level feature representations, an almost necessary step for improving learning in the presence of small datasets. Using this baseline, the CNNs were able to learn low level features. This learning was transferred during training with the mosquito image dataset for composing higher level features for our target task of species identification.

### Hardware requirements

The hardware configuration for the experiments included a workstation equipped with 64*G* of memory, 2 Intel Xeon E5 Computer Processing Units (CPUs) at 2.10GHz, and 4 Pascal Titan X Graphics Processing Units (GPUs). GPUs are widely used in deep learning applications to accelerate training and inference tasks. On average, training a network on our image dataset took 90 minutes using all four GPUs. In the final phase, when the trained CNN was presented with the test set to predict the class of mosquito images, the average time per individual image inference using 1 GPU is 1.19 seconds per batch of 263 images. This is approximately equivalent to processing 221 images per second. Although GPUs are preferable, it is possible to test the CNN for image class prediction (i.e. species) with only CPUs. When the GPU is disabled and only the CPUs are used, the average time is 34.07 seconds for a batch of 263 images, equivalent to 8 images per second.

## Results

The first goal of our experiments was to train a CNN to distinguish each of the 17 classes in our dataset, that is, categorizing images of different genus, species, cryptic species within the *An*. *gambiae* complex, two strains of *An*. *gambiae* s.s., and two storage methods of *An*. *stephensi*. Following our training protocol, the first step was to select the best architecture. Table 3 shows the respective training and validation accuracies for our selected architectures and varying their number of layers.

**Table 3:** Overall training and validation accuracies for different CNN architectures with variable numbers of layers.

Architecture	# of Layers	Train Acc.	Validation Acc.
ResNet	18	99.10%	92.02%
	34	99.01%	93.16%
	50	98.43%	90.87%
	101	99.46%	95.44%
	152	99.31%	93.54%
Wide ResNet	50	99.28%	93.92%
	101	99.48%	93.92%
ResNext	50	98.77%	94.30%
	101	99.37%	95.82%
DenseNet	121	99.09%	92.02%
	161	99.50%	94.30%
	169	99.50%	95.06%
	201	99.88%	**96.20%**

The DenseNet with 201 layers (DenseNet-201) presented the highest validation accuracy at 96.20%. We subsequently trained several versions of this architecture using different batch sizes in the range of [2^4^, 2^7^]. Decreasing the batch size did not increase the highest validation accuracy. A batch size of 128 images yielded the highest validation accuracy. Next, the Densenet-201 with a batch-size of 128 was trained with different optimizers and learning rates. These included AdaGrad, RMSProp, Adam, Stochastic Gradient Descent (SGD), and ASGD. The final accuracies for each optimizer are summarized in Table 4.

**Table 4:** Overall training and validation accuracies of a DenseNet with 201, varying the selected optimizer. For each optimizer, accuracies of the best learning rate are reported.

Optimizer	Train	Validation
AdaGrad	99.93%	96.96%
RMSProp	99.83%	96.20%
Adam	99.95%	96.96%
SGD	99.98%	96.96%
ASGD	100%	**97.34%**

After employing an optimizer and fine tuning the learning rate, our highest accuracy overall was 97.34%, achieved using Densenet-201, trained with a batch-size of 128, with a ASGD optimizer, and a learning rate of 2^−2^.

We evaluated the accuracy of our highest accuracy configuration using the test set of images, which were withheld from training and validation. When presented with the test set, this CNN configuration yielded an accuracy of **96.96%**. Among the seven misclassifications, two were between *An*. *arabiensis* and *An*. *coluzii*, species within the *An*. *gambiae* complex known to have cryptic morphological variation. Two misclassifications occurred with the desiccated *An*. *stephensi* and members of the *An*. *gambiae* complex. There was no confusion between the classes of images of dried versus flash frozen *An*. *stephensi*. The detailed confusion matrix in Table 5 summarizes CNN predictions for images in the test set.

**Table 5:** Confusion matrix for the 17 categories of the held-out set of images using the best hyperparameter configuration. Rows and columns represent predicted and actual classes respectively.

	(1)	(2)	(3)	(4)	(5)	(6)	(7)	(8)	(9)	(10)	(11)	(12)	(13)	(14)	(15)	(16)	(17)
*An*. *arabiensis* * (1)	**28**	2	0	0	0	0	0	0	0	0	0	0	0	0	0	0	0
*An*. *coluzzii* * (2)	0	**10**	0	0	0	0	0	0	0	0	0	0	0	0	0	0	0
*An*. *gambiae* * (3)	0	0	**17**	0	0	1	0	0	0	0	0	0	0	0	0	0	0
*An*. *gambiae* * (4)	0	0	0	**8**	0	0	0	0	0	0	0	0	0	0	0	0	0
*An*. *merus* * (5)	0	0	0	0	**7**	0	0	0	0	0	0	0	0	0	0	0	0
*An*. *funestus* (6)	0	0	0	0	1	**20**	0	0	0	0	0	0	0	0	0	0	0
*An*. *faurati* (7)	0	0	0	0	0	0	**9**	0	0	0	0	0	0	0	0	0	0
*An*. *minimus* (8)	0	0	0	0	0	0	0	**18**	0	0	0	0	0	0	0	1	0
*An*. *stephensi* (dried) (9)	1	0	1	0	0	0	0	0	**25**	0	0	0	0	0	0	0	0
*An*. *stephensi* (10)	0	0	0	0	0	0	0	0	0	**8**	0	0	0	0	0	0	0
*An*. *albimanus* (11)	0	0	0	0	0	0	0	0	0	0	**9**	0	0	0	0	0	0
*An*. *atroparvus* (12)	0	0	0	0	0	0	0	0	0	0	1	**6**	0	0	0	0	0
*An*. *freeborni* (13)	0	0	0	0	0	0	0	0	0	0	0	0	**15**	0	0	0	0
*An*. *quadrimaculatus* (14)	0	0	0	0	0	0	0	0	0	0	0	0	0	**8**	0	0	0
*Cu*. *quinquefasciatus* (15)	0	0	0	0	0	0	0	0	0	0	0	0	0	0	**8**	0	0
*Cu*. *tarsalis* (16)	0	0	0	0	0	0	0	0	0	0	0	0	0	0	0	**26**	0
*Ae*. *aegypti* (17)	0	0	0	0	0	0	0	0	0	0	0	0	0	0	0	0	**33**

(*) indicates that the species is a member of the *Anopheles gambiae* species complex.

### Effect of data augmentation

The pre-processing of images using data augmentation was critical in order to achieve the high degree of accuracy in the 17-way classification task. To illustrate the importance of data augmentation we repeated these protocols in the absence of data augmentation, but with all other protocols remaining the same. Removing this step of the process resulted in a final accuracy in prediction of test set images of 70.34%, a 26.62% drop. Despite this drop, the prediction accuracy of the trained CNN on mosquito images without data augmentation still surpasses the human eye in delimiting cryptic species and strains within *An*. *gambiae* complex. However, the inclusion of data augmentation results in a CNN that can be applied with a degree of confidence in the results that approaches the standard methods of identification using molecular markers by PCR.

### Species classification of female mosquitoes

As only female mosquitoes seek blood for reproduction, in practice species identification of human malaria vectors excludes males. Taxonomic keys of adult mosquito vector species focus on morphological characteristics of female specimens [9, 50]. Sorting and removing males is part of the time consuming process of visual species identification in field surveillance settings. To create an automatic classification tool for future applications in a field setting, we repeated the training protocols for only images of female mosquitoes. Sex determination of adult mosquitoes prior to imaging was carried out by a trained medical entomologist. Here again, the best configuration after training and validation was a DenseNet CNN with 201 layers, a batch-size of 128, and using the ASGD optimizer with a learning rate of 2^−3^. A model trained with this configuration reached an accuracy of 95.24% with images from the test set. The exclusion of images with male mosquitoes therefore resulted in only a modest drop in the overall accuracy achieved, dropping only 1.72%. Of the eight misclassifications, four occurred between the species within the *An*. *gambiae* complex. The detailed CNN predictions for the test set of only female images are summarized in the confusion matrix in Table 6.

**Table 6:** Confusion matrix for the 17 categories of the held-out set of images using the best hyperparameter configuration. Rows and columns represent predicted and actual classes respectively. Note that images of male specimens were excluded from this experiment.

	(1)	(2)	(3)	(4)	(5)	(6)	(7)	(8)	(9)	(10)	(11)	(12)	(13)	(14)	(15)	(16)	(17)
*An*. *arabiensis* * (1)	**17**	1	0	1	0	0	0	0	0	0	0	0	0	0	0	0	0
*An*. *coluzzii* * (2)	1	**7**	0	0	0	0	0	0	0	0	0	0	0	0	0	0	0
*An*. *gambiae* * (3)	1	0	**11**	0	0	0	0	1	0	0	0	0	0	0	0	0	0
*An*. *gambiae* * (4)	0	0	0	**4**	0	0	0	0	0	0	0	0	0	0	0	0	0
*An*. *merus* * (5)	0	0	0	0	**4**	0	0	0	0	0	0	0	0	0	0	0	0
*An*. *funestus* (6)	0	0	0	0	0	**12**	0	0	0	0	0	0	0	0	0	0	0
*An*. *faurati* (7)	0	0	0	0	0	0	**1**	0	0	0	0	0	0	0	0	0	0
*An*. *minimus* (8)	0	0	1	0	0	0	0	**7**	0	0	0	0	0	0	0	0	0
*An*. *stephensi* (dried) (9)	0	0	0	0	0	0	0	1	**16**	0	0	0	0	0	0	0	0
*An*. *stephensi* (10)	0	0	0	0	0	0	0	0	0	**4**	0	0	0	0	0	0	0
*An*. *albimanus* (11)	0	0	0	0	0	0	0	0	0	0	**6**	0	0	0	0	0	0
*An*. *atroparvus* (12)	0	0	0	0	0	0	1	0	0	0	0	**6**	0	0	0	0	0
*An*. *freeborni* (13)	0	0	0	0	0	0	0	0	0	0	0	0	**7**	0	0	0	0
*An*. *quadrimaculatus* (14)	0	0	0	0	0	0	0	0	0	0	0	0	0	**7**	0	0	0
*Cu*. *quinquefasciatus* (15)	0	0	0	0	0	0	0	0	0	0	0	0	0	0	**8**	0	0
*Cu*. *tarsalis* (16)	0	0	0	0	0	0	0	0	0	0	0	0	0	0	0	**20**	0
*Ae*. *aegypti* (17)	0	0	0	0	0	0	0	0	0	0	0	0	0	0	0	0	**23**

(*) symbolizes species is a member of the *Anopheles gambiae* species complex.

### Dual prediction of mosquito species and sex

We trained a network with an additional output that predicts the sex of the mosquito in the image. Under these settings, the CNN receives an input image and predicts one of the 17 categories, and also an additional output that indicates whether the input image contains a male or female specimen. Incorporating the second task of predicting mosquito sex does not sacrifice prediction power for class (i.e. species or strain). Training a single network to perform this dual task, achieves 96.95% accuracy on the entire test set to predict the correct class, and 98.48% accuracy to predict the correct sex of the input image. While, in practice, determining the sex of adult mosquitoes is a relatively simple visual task for humans it still requires workforce training and labor. These results indicate that this step in the mosquito identification process can also become automated with mosquito imaging with a high degree of accuracy.

### Interpreting the CNN probabilistic output

**Fig 6:**
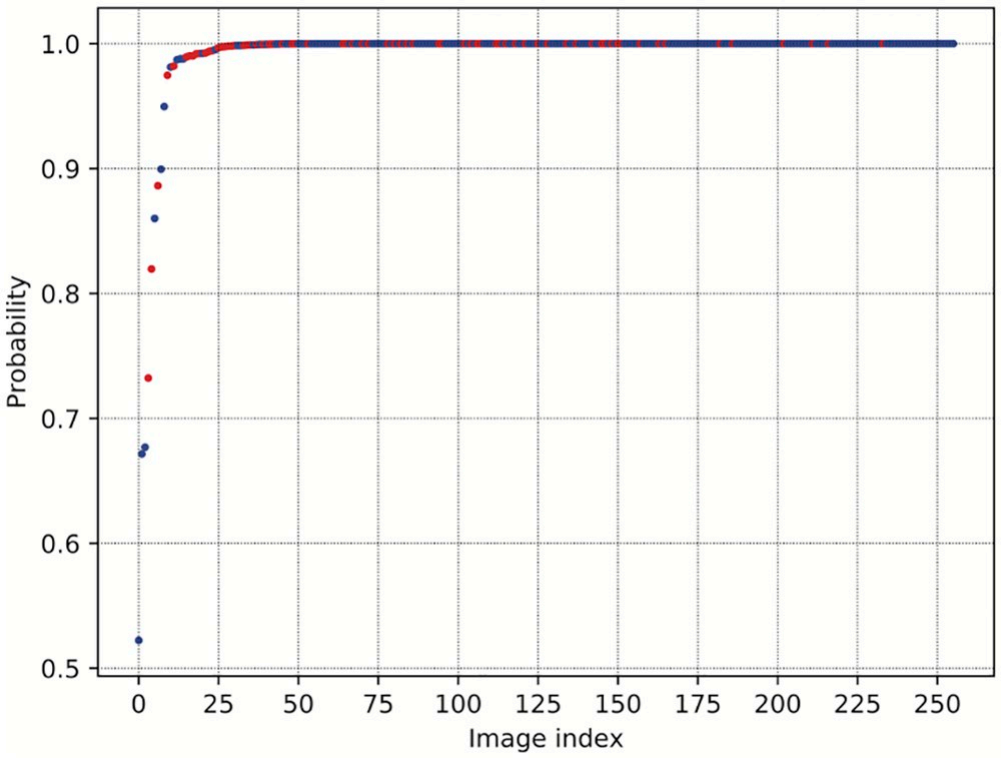
Highest predicted probabilities for true positives. Blue dots represent probabilities for each of the images that were correctly classified in the test set. Red dots represent images from the member species of the *Anopheles gambiae* complex.

The output of the last layer of a CNN is a set of probabilities. That is, with 17 classes and for every input image, the network provides 17 values. Each value is a probability that the input image belongs to one of the classes. For each image, these sets of probabilities always add up to 1, such that they can also be interpreted as probability distributions. The output of the predicted class label is determined by selecting the highest probability value produced by the last layer. Further, a higher the probability for the *winner* class can be interpreted as a more confident prediction by the network. In comparing the highest probabilities of all the images of the test set we can interpret the overall confidence of prediction for the CNN. In Fig 6 we plot the highest predicted probabilities for each of the test set images that were correctly classified. To facilitate interpretation they have been sorted by probability from lowest to highest. We found only for 7 specimen images had probability scores that fell below 0.9. This underscores that the trained and validated CNN has a high confidence in test set predictions overall. Notably, this result holds even with the inclusion of species with cryptic morphological variation and strains of a single species, as indicated by the red dots in Fig 6.

### Visualizing the CNN feature space

For the CNN trained with the best configuration of hyperparameters, we collected the output from the last convolutional layer of the network. The output was considered after feeding the CNN with *all* the images in our original dataset, i.e. including training, validation, and test sets. When an image is passed through the network, the output of this layer is a 1, 920-dimensional array of values. This array can be interpreted as a feature extractor.

**Fig 7:**
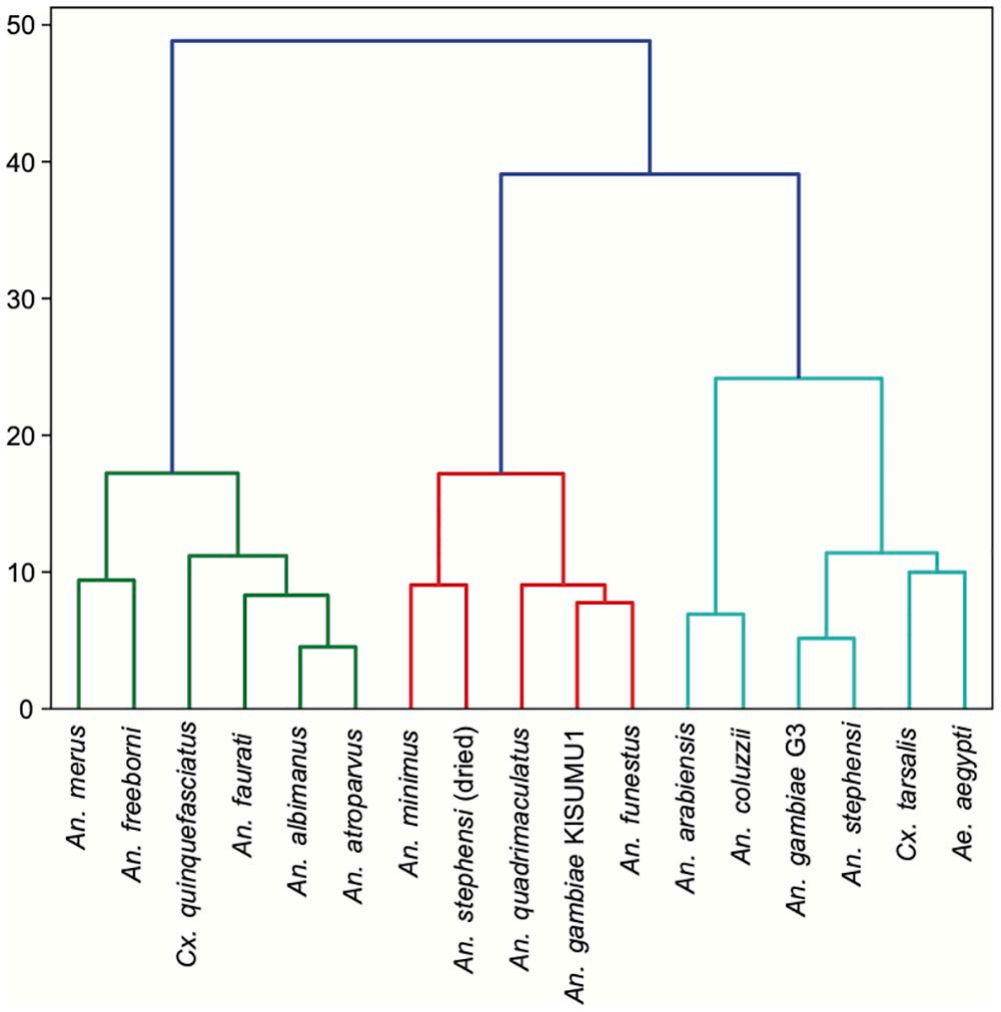
Clustering feature representations. Resulting dendrogram after applying Hierarchical Agglomerative Clustering. Colors indicate major branches.

We applied Hierarchical Agglomerative Clustering (HAC) to the set of feature arrays and generated a dendrogram shown in Fig 7 to represent feature distance relationships in the same manner as cladograms, but without a direct biological meaning. Indeed, the KISUMU1 and G3 strains of *An*. *gambiae* s.s. (classes 3 and 4 respectively) are more distantly related based on the feature output array than *An*. *gambiae* G3 with either *An*. *arabiensis* or *An*. *coluzzii*.

**Fig 8:**
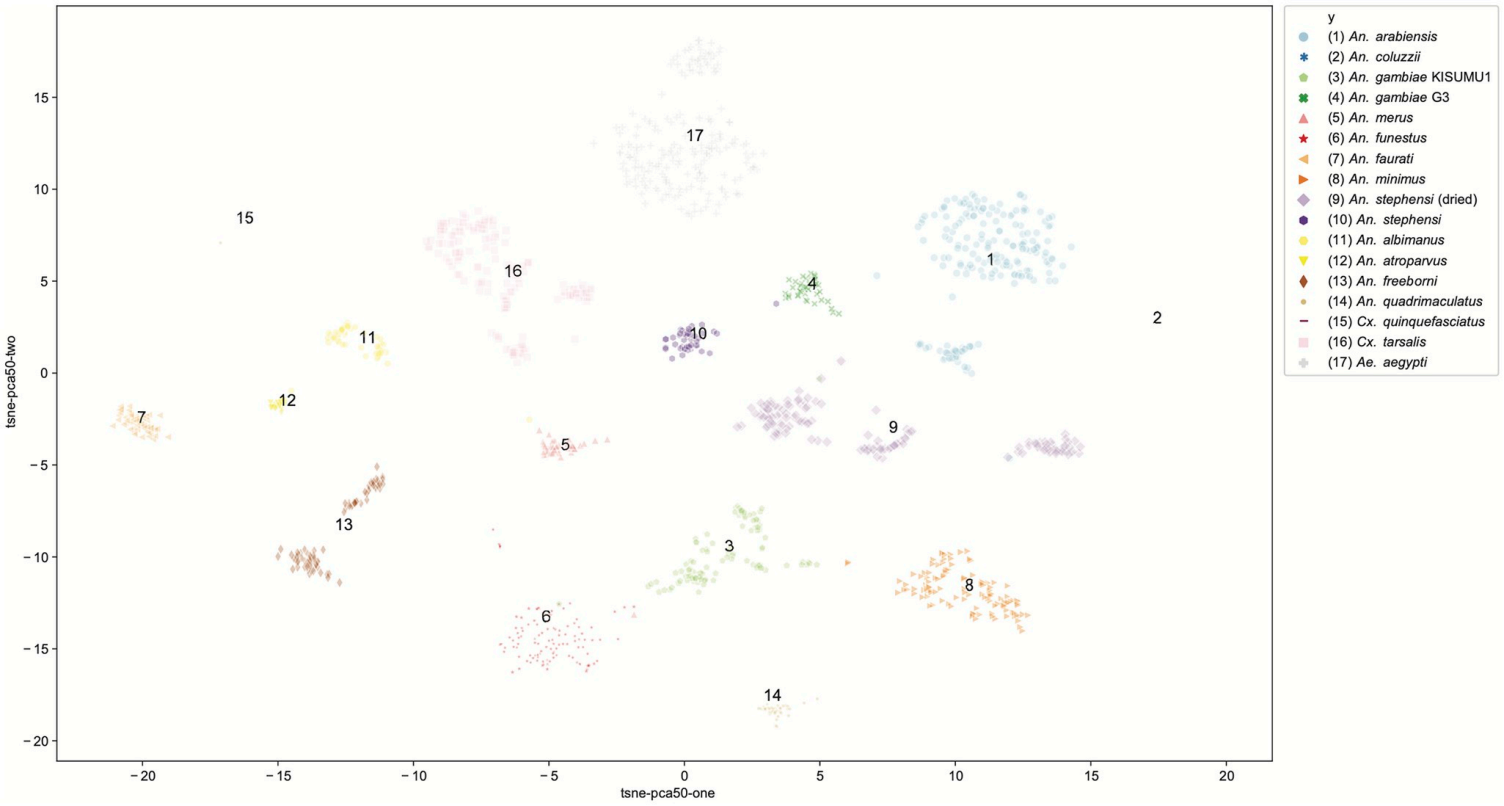
Visualizing the feature space. t-SNE projection applied to the features extracted from the last convolutional layer. Colors are used to denote different clusters.

Using a combination of Principal Components Analysis (PCA) and t-Distributed Stochastic Neighbor Embedding [51], we visualized all of the images in a 2-dimensional projected space of features. This is shown in Fig 8, where output clusters are labeled with class label and assigned a color. Each point is an image, such that the larger the cloud of points in a cluster the more variability between the images within the class. The distance between clusters may be interpreted as the morphological difference between classes in this feature space. Clusters were easily distinguished, indicating low numbers of misclassifications. While the units of this feature space are difficult to interpret, the distances between clusters may be useful to compare the CNN to actual biological relationships between the morphological characteristics of the mosquito genera, species, and strains analyzed.

## Discussion

CNNs are powerful methods for distinguishing classes of images, and there is a growing interest in applying these methods to delimit species [31, 32]. However, very few studies have applied deep learning to distinguish species with cryptic morphological variation. Here, we train, validate, and test CNNs to predict genus, species, and strains of mosquitoes that transmit human pathogens, with an emphasis on malaria vectors and cryptic species in the *An*. *gambiae* complex. These results are both a proof-of-concept that CNNs can be applied to distinguish species with cryptic variation, and a case study in methods implementing deep learning for mosquito identification. Our results indicate that data augmentation dramatically increases prediction capabilities, overcoming a common difficulty of limited sizes of image datasets. We demonstrate that not only can up to 96.96% accuracy in species identification be achieved, the incorporation of dual tasks of species *and* sex identification does not decrease the prediction accuracy of the CNN. These results are promising for future applications of CNNs on mosquito species images at a regional or local scale to automate species identification in mosquito surveillance for malaria and other mosquito-borne diseases.

### Deep learning to distinguish cryptic species

Few studies have applied deep learning to distinguish species with cryptic morphological variation, and our results indicate deep learning can exceed human visual capacity to distinguish species and strains of *Anopheles* based on morphological characters. Such applications may be more broadly applied. Deep learning was used to recognize New Zealand plant and moth species characterized in the study as ‘difficult to identify,’ achieving approximately 74% accuracy for plants in the genus Corposoma, and 87% accuracy for 11 species of moth [52], though little information is provided regarding the morphological variation in these groups. Among malaria mosquito species, only one study has applied machine learning methods to distinguish between *An*. *gambiae* and *An*. *arabiensis* [41]. In this work, five different machine learning methods were trained and evaluated on vibrational spectroscopy in the mid-infrared region to distinguish two species *An*. *gambiae* and *An*. *arabiensis*, with a maximum identification accuracy of 82.6% after applying boot-strapping and combining, by bagging, the best 10 trained models [41].

With deep learning, we exceed these prediction accuracies and increase the number of classes to include 15 mosquito vector species, including 4 member species of the *An*. *gambiae* complex. Studies applying deep learning to species identification questions often face the challenge of limited image datasets. Datasets with large numbers of images per species are not readily available for most animals and plants. Without data augmentation the testing accuracy of our best performing CNN dropped by 26.32%. The data augmentation methods presented here required minimal pre-processing of images, and increased the size of the training set by 48 times. When carefully employed in only training sets to avoid pseudoreplication, data augmentation can drastically improve the prediction results of the CNN.

That the DenseNet-201 was able to distinguish genera, species, species with cryptic morphological variation, as well as 2 strains *An*. *gambiae* s.s. suggests that CNNs are a potential tool in the study of morphological divergence between populations. By including images of specimens from a single colony using different storage methods we sought to further test the boundaries of delimitation with deep learning. The ability of the DenseNet-201 to accurately predict classes which included dried versus freshly frozen *An*. *stephensi* from the colony highlights the importance of consistent methods in specimen collection, storage, and image capture (see Fig 1). Given the ability of the CNN to accurately delimit the classes separated only by storage method, we caution against applying CNNs developed from dried museum specimens to make identification predictions of fresh field collected mosquito specimens.

### CNN feature space and prediction visualizations

In order to peek into the ‘black box’ of the CNN architecture and predictions we employed a variety of visualizations. One criticism of neural networks approaches is that the features of images that drive the learning process are difficult to identify. Fig 8 shows a visualization of the feature space of the entire dataset, providing some explanation of the CNN architecture. Because the image diversity (i.e. positioning, magnification, depth of focus) was stratified deliberately to be consistent across classes, variability in feature space may be understood as morphological variability between specimens. Despite being the same species, the two strains *An*. *gambiae* s.s., G3 and KISUMU1, appear more closely clustered in feature space with other *Anopheles* species than with each other. Genetic relationships between species is not readily interpretable with this approach, but the variability of images within a class can be considered from a biological standpoint. For example, *An*. *gambiae* G3 is known to be polymorphic, mongrel strain. Based on the clustering visualizations (Figs 7 and 8), there is more variability in feature parameter space in this strain than in the *An*. *gambiae* KISUMU1 strain.

**Fig 9:**
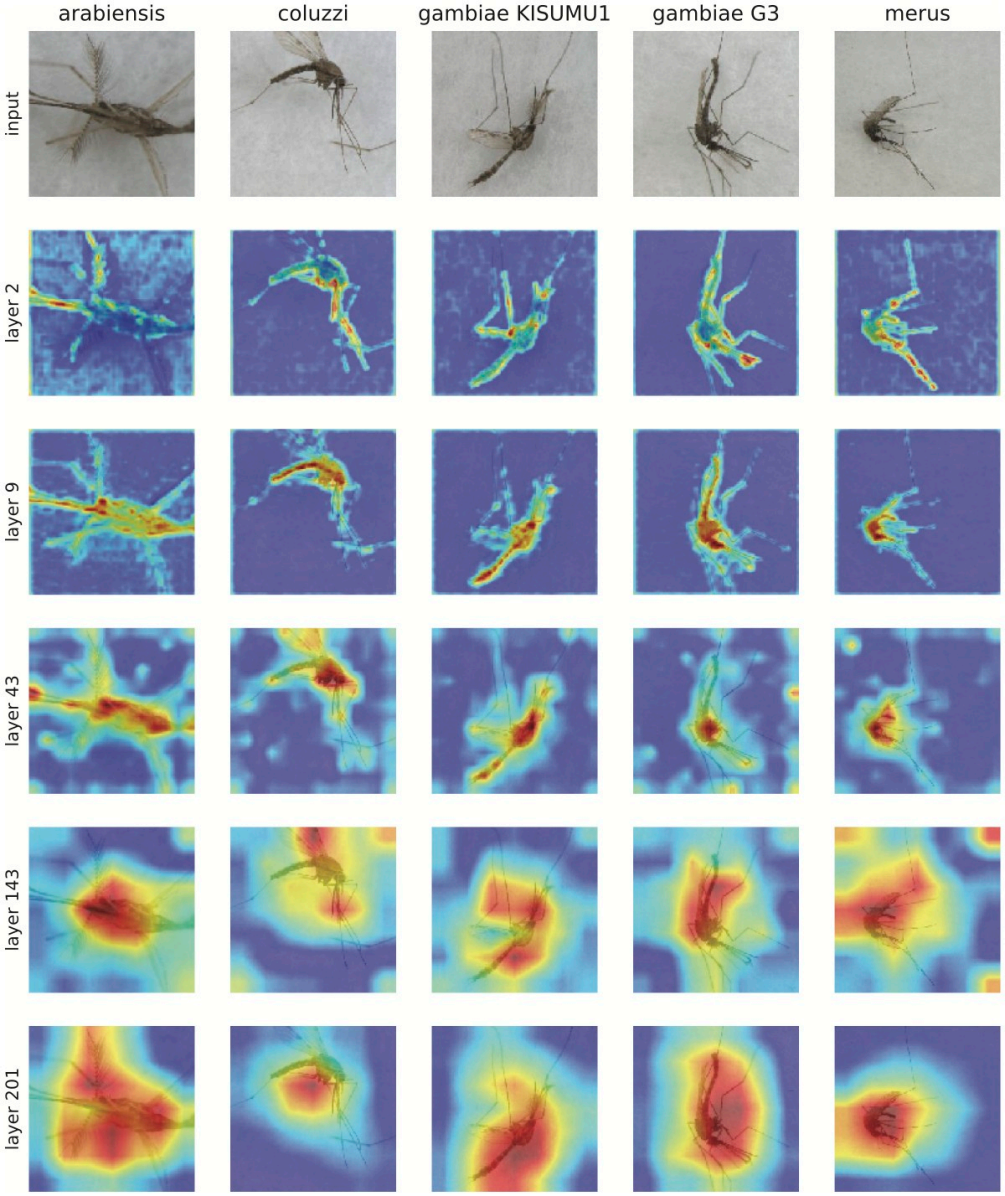
Applying Grad-Cam++ to test images. Five images from the were selected from the test set to be analyzed with Grad-Cam++. This is a visualization method for identifying the regions in the image that can explain the final classification made by the network.

In addition, recent developments in visualization methods for CNNs [53], provide interesting visual clues to the basis for the CNN predictions as learning progresses through the neural network layers. Using Grad-Cam++ we visualized and identified the regions in the image that can explain the final classification made by the network. Approaches like this provide a relative measure of importance to each pixel in an image. Fig 9 shows activations of several intermediate layers from our best model as applied to 5 arbitrary test images from the *Anopheles gambiae* complex. Red and yellow areas in the activation maps depict regions where the neural network places its attention more heavily when calculating the prediction. Early layers appear to focus on the proboscis, wings, legs, and limited portions of the abdomen. The visualization in the early layers correlates well with standard human visual identification methods often used to distinguish African *Anopheles* species [9]. As layers increase, the range of pixels weighted heavily into the CNN model expands to the head, thorax, and abdomen in all 5 images, shifting to the wings in some, expanding to the entire body including legs, which broadens the area of pixels in red and yellow. While these images can be helpful to provide some explanation of the CNN architecture, by the final layer it is difficult to interpret beyond recognizing that the CNN is basing predictions on the mosquito rather than background.

### Public health applications for malaria surveillance

Our results indicate that deep learning can improve our ability to morphologically distinguish among members of the *An*. *gambiae* complex species complex, going beyond methods currently available using taxonomic identification keys for *An*. *gambiae* s.l. [9, 50]. We demonstrated as a proof-of-concept that malaria mosquito vector species and sex identification can be automated using whole-body images. The methods of image capture, image processing, and deep learning techniques presented here provide an example pipeline methodology. The code for training, validating, and testing a CNN as well as the malaria mosquito image dataset are made available through Github and Dryad respectively to facilitate future training and validation of CNNs in applied settings with additional mosquito image datasets.

Currently, species within the *An*. *gambiae* complex are distinguished by PCR using molecular markers. While these methods are the current gold standard, there are limitations to PCR identification, particularly if non-vector species are included in assays [7]. Additional training of the DenseNet-201 model with images of wild-populations is one application of these study results, and would provide an independent avenue to identify species as a comparison to PCR. Because the imaging process is non-destructive for mosquito specimens, both computer vision methods and molecular methods can be employed in parallel for malaria and malaria vector surveillance. Moreover, several genera within Culicidae that transmit human pathogens contain species complexes with cryptic morphological variation, expanding the potential applications of this method to improve mosquito-borne disease surveillance for other pathogens.

The equipment needed to incorporate these methods are microscope and camera for image capture and a CPU. Although the addition of GPUs may provide faster inference times, they are not required. The results of this study demonstrate that rapid and accurate automatic identification of malaria vector species, including those with cryptic morphologies, is possible with CNNs. Further investigation is warranted to determine how best to implement these methods in a field setting and in coordination with existing mosquito surveillance programs.

## Conclusion

The development of an independent and accurate method of species identification can potentially improve mosquito surveillance practices. Further, these methods open doors for people with visual, spatial, or motor-control impairments to engage in mosquito surveillance and the study of mosquito vectors. Finally, we demonstrate that deep learning can be applied to distinguish cryptic species and strains within a species. This is an ongoing barrier to effective surveillance of human malaria vectors in areas where cryptic species occur in sympatry. The methods and findings presented provide a foundation for the development of deep learning tools for rapid and accurate species identification of malaria mosquito vectors. Computer vision classification of images with deep learning may be broadly applicable as a non-destructive technique to morphologically distinguish cryptic species and populations.
